# Neuroprotective pentapeptide CN-105 improves functional and histological outcomes in a murine model of intracerebral hemorrhage

**DOI:** 10.1038/srep34834

**Published:** 2016-10-07

**Authors:** Beilei Lei, Michael L. James, Ji Liu, Guanen Zhou, Talaignair N. Venkatraman, Christopher D. Lascola, Shawn K. Acheson, Laura G. Dubois, Daniel T. Laskowitz, Haichen Wang

**Affiliations:** 1 Department of Anesthesiology, Duke University Medical Center, Durham, NC 27710, USA; 2 Department of Neurology, Duke University Medical Center, Durham, NC 27710, USA; 3 Department of Neurology, Huanhu Hospital, Tianjin, 300060, China; 4 Department of Radiology, Duke University Medical Center, Durham, NC 27710, USA; 5 Department of Psychiatry, Duke University Medical Center, Durham, NC 27710, USA; 6 Neurobiology Research Lab, Durham VA Medical Center, Durham, NC 27705, USA; 7 Proteomics and Metabolomics Shared Resource, Duke University, Durham, NC 27710, USA

## Abstract

Presently, no pharmacological treatments have been demonstrated to improve long-term functional outcomes following intracerebral hemorrhage (ICH). Clinical evidence associates apolipoprotein E (apoE) genotype with ICH incidence and outcome. While apoE modifies neuroinflammatory responses through its adaptive role in glial downregulation, intact apoE holoprotein is too large to cross the blood-brain barrier (BBB). Therefore, we developed a 5-amino acid peptide – CN-105 – that mimics the polar face of the apoE helical domain involved in receptor interactions. In the current study, we investigated the therapeutic potential of CN-105 in a mouse model of ICH. Three doses of CN-105 (0.05 mg/kg) was administered by tail vein injection within 24 hours after ICH induction. Functional assessment showed durable improvement in vestibulomotor performance after CN-105 treatment, as quantified by increased Rotarod latencies on Days 1–5 post-ICH, and long-term improvement in neurocognitive performance, as quantified by reduced Morris water maze latencies on Days 29–32 post-ICH. Further, brain water content was significantly reduced, neuroinflammation was decreased and hippocampal CA3 neuronal survival was increased, although hemorrhage volume was not affected by CN-105. We concluded, therefore, that pentapeptide CN-105 improved short- and long-term neurobehavioral outcomes in a murine model of ICH, suggesting therapeutic potential for patients with acute ICH.

Primary intracerebral hemorrhage (ICH) affects as many as 80,000 people every year in the United States alone. It is associated with poor neurological outcome, with less than one third of survivors returning to functional independence^1^. Sadly, little progress has been made over the last 20 years to improve recovery after ICH^2^. Brain inflammatory responses to ICH mediate the development of cerebral edema, intracranial hypertension, and secondary neuronal injury. Thus, pharmacological intervention that reduces neuroinflammation, cerebral edema, and secondary tissue injury would be a significant finding.

Genetic links to ICH outcomes may provide a crucial link to the development of effective therapeutic strategies. For example, apolipoprotein E (apoE) genotype has been repeatedly associated with ICH incidence and outcome. ApoE is a multifunctional 299-amino acid protein, and the primary apolipoprotein in the central nervous system (CNS), with 3 common human isoforms: apoE2, apoE3, and apoE4. Several studies have shown that carriers of the apoE4 allele have much higher mortality rates and poorer neurological recovery after ICH^3^,^4^,^5^,^6^,^7^. In earlier work, an association was found between the apoE4 isoform, and increased perihematomal edema and poorer outcome in ICH patients^7^. Further, in a preclinical model of ICH, targeted replacement “knock-in” mice bearing the human apoE4 allele, showed increased cerebral edema and neuroinflammation with worsened neurobehavioral outcomes compared to apoE3 mice^8^,^9^.

Thus, modifying the effects of endogenous apoE may be an effective therapeutic strategy for mitigating the neuroinflammatory response to ICH. Notably, in several preclinical models of acute brain injury, including ICH, exogenous apoE-mimetic peptides improved functional and histological outcomes when administered intravenously after injury^8^,^9^,^10^,^11^,^12^,^13^,^14^,^15^. Moreover, these peptides are well tolerated, cross into the CNS compartment after intravenous administration, and reduce neuroinflammation after acute brain injury^14^,^16^,^17^. Recently, a 5-amino acid apoE-mimetic peptide – CN-105 (Ac- VSRRR-amide) – was designed by linearizing the receptor binding face of the apoE alpha helix. The present study evaluated the therapeutic efficacy of this pentapeptide in our murine model of collagenase-induced ICH. We hypothesized that intravenous administration of CN-105 in this model reduces cerebral edema, increases neuronal survival, and improves vestibulomotor and cognitive performance after experimental ICH.

## Results

### Improved Pharmacokinetics with Neuroprotective Pentamers

**Figure 1:**
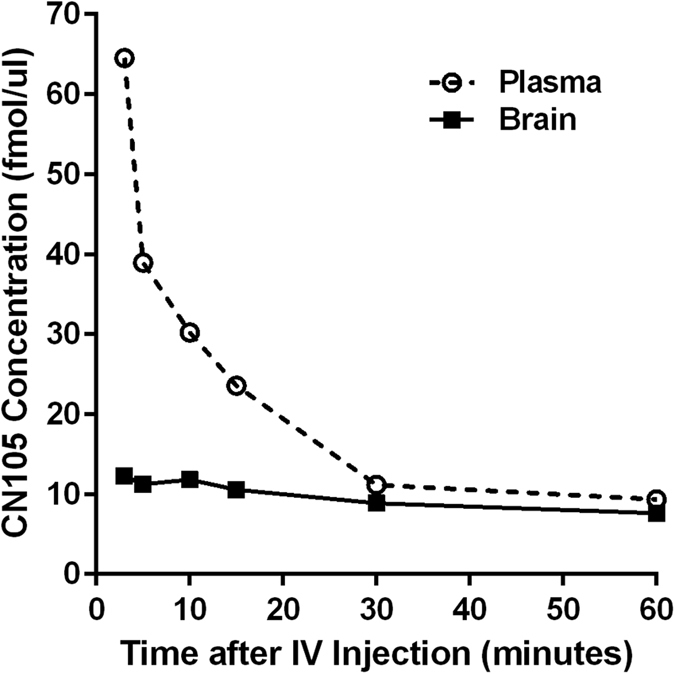
Brain and plasma pharmacokinetic analysis of CN105 demonstrated a terminal plasma half-life of 27 minutes, with AUC_0–60_ in plasma of 1253 min*fmol/*u*l, and in brain 548 min*fmol/*u*l.

One rationale for creating the smaller apoE mimetic peptides was to enhance CNS penetration and possibility of noninvasive mechanisms of delivery (for example, intranasal). To define pharmacokinetic parameters, we performed initial non-GLP studies in mice using stable radioisotope labeled CN-105 peptide (MW of heavy CN-105 was 362.7 vs. 357.7). Our analysis demonstrated a terminal plasma half-life of 27 minutes, with AUC_0–60_ in plasma of 1253 min*fmol/*u*l, and in brain 548 min*fmol/*u*l (Fig. 1). The total CNS exposure was 44%; of note, this is approximately 5 fold higher than prior apoE based mimetic peptides^15^.

### Hemorrhage volume was not changed after CN-105 treatment

**Figure 2:**
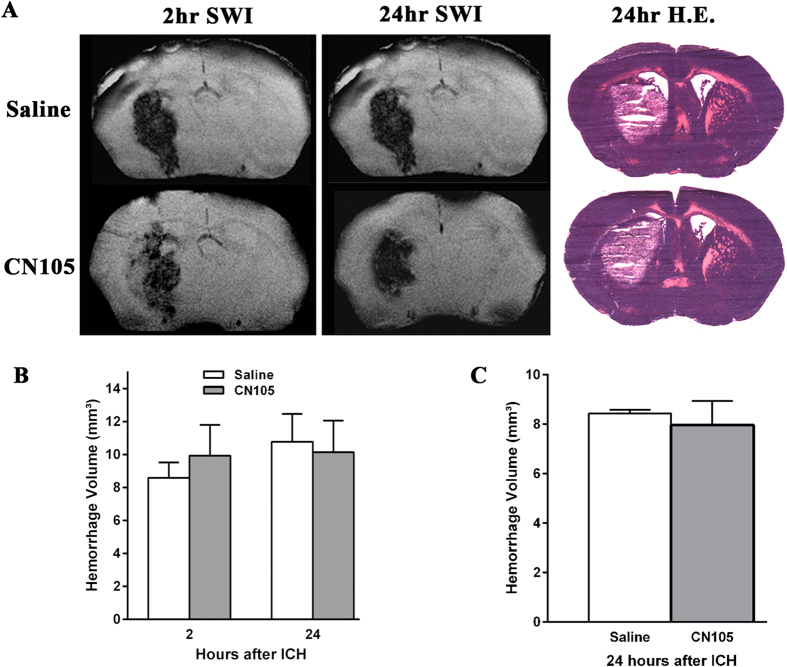
Hemorrhage volume was similar between CN-105- and saline-treated mice. CN-105 did not reduce hemorrhage volume as assessed by SWI -MRI (**A,B**) images or by H&E staining (**A,C**). (n = 3–4/group).

Both Magnetic resonance imaging (MRI) and traditional Hematoxylin and Eosin (H&E) staining were used to measure hemorrhage volume in brains after ICH. No difference in hematoma volume between CN-105 and vehicle-treated animals was detected by MRI at 2 hours after ICH induction (prior to treatment) (9.93 ± 1.88 mm^3^ vs 8.59 ± 0.93 mm^3^, p = 0.59), indicating that animals in both groups received similar injury (Fig. 2A,B). SWI MRI data also revealed no evolution of hemorrhage volume between 2 and 24 hours post ICH (8.59 ± 0.93 mm^3^ vs 10.77 ± 1.70 mm^3^, p = 0.31), and no effect of CN-105 treatment compared to saline group (10.14 ± 1.92 mm^3^ vs 10.77 ± 1.70 mm^3^, p = 0.82) (Fig. 2A,B). Similarly, H&E staining showed no difference in hemorrhage volume after CN-105 treatment vs saline treatment at 24 hours after ICH (8.43 ± 0.15 mm^3^ vs 7.97 ± 0.97 mm^3^, p = 0.66) (Fig. 2A,C).

### Brain water content was reduced in CN-105 mice

**Figure 3:**
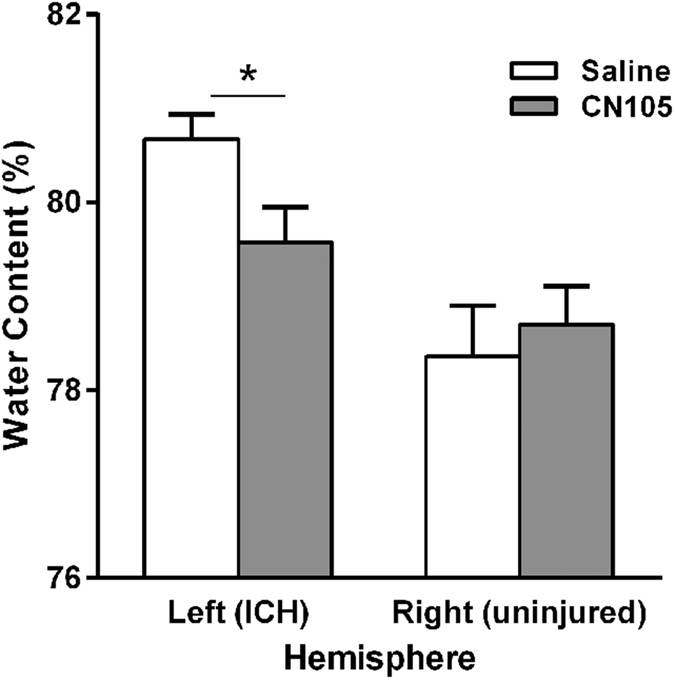
Brain water content was reduced in CN-105 mice at 24 hours post ICH. (*p < 0.05; n = 6–8/group).

Brain water content^18^ was higher in the injured hemisphere among vehicle-treated animals compared to CN-105-treated animals (80.67 ± 0.26% vs 79.57 ± 0.38%, p = 0.029) at 24 hours after injury. No such treatment effect was observed in the contralateral hemisphere (78.36 ± 0.54% vs 78.70 ± 0.41%, p = 0.64). (Fig. 3).

### Functional outcomes were improved in CN-105 treated mice

Given the reduction in cerebral edema when the first and second doses was administered at 2 and 6 hours after ICH, we next evaluated the possibility that the therapeutic window could be extended to administer the first and second doses at 4 and 8 hours after ICH, as this would facilitate translation to a clinical trial. The third dose are all given at 24 hours after ICH.

**Figure 4:**
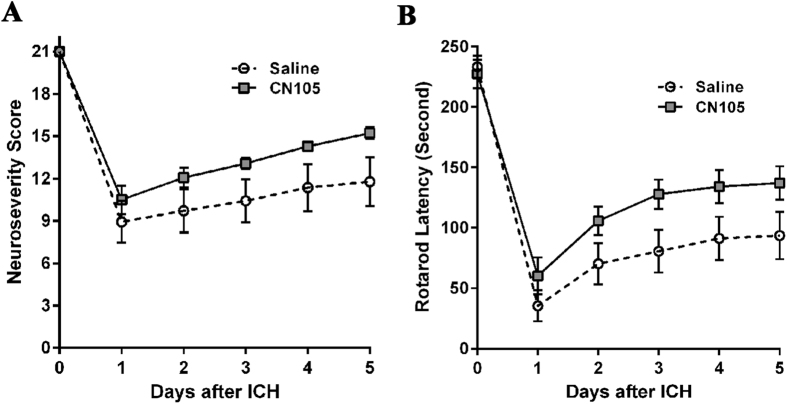
Short-term function improved in CN-105 animals over 5 days following ICH. CN-105 mildly, but not significantly, improved recovery of neurobehavioral function (**A**). CN-105 significantly improved recovery of vestibulomotor function, based on rotarod testing (**B**). (n = 14/group).

All animals demonstrated maximum neuroseverity scores^8^ of 21 prior to ICH. Analysis of neuroseverity score data following ICH indicated that animals in the CN-105- and vehicle-treated groups demonstrated equivalent neurobehavioral impairment on Day 1 following ICH (p = 0.71). Between Day 1 and Day 5 after ICH, neuroseverity scores in the CN-105-treated group showed mild but not significant improvement in neurobehavioral function compared to the vehicle group (p = 0.12) (Fig. 4A).

Rotarod testing^19^ showed significantly improved motor functional recovery in CN-105 mice compared to vehicle mice (p = 0.0435) (Fig. 4B).

**Figure 5:**
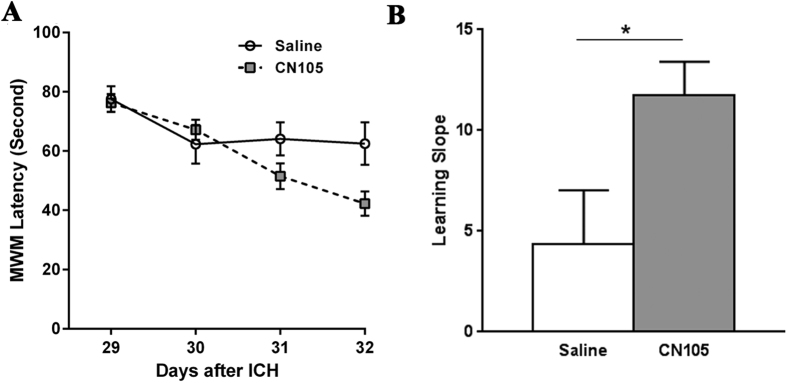
Neurocognitive function showed long-term improvement in CN-105 mice, based on MWM testing. (**A**) Latencies in CN-105-treated mice were lower and learning curves were steeper; (**B**) slope of learning curve presented as absolute value) over 4 days of testing, compared to saline-treated group. (*p < 0.05; n = 14/group).

Escape latencies in the Morris Water Maze (MWM)^20^ tests were not significantly different in CN-105- vs vehicle-treated animals on the first day of water maze test (p = 0.4). However, over Days 29–32 after ICH, CN-105 mice had significantly lower latencies vs the vehicle group (Day x Group: F(3,66) = 3.61, p = 0.02) (Fig. 5A). Moreover, the CN-105 group had significantly steeper learning curves with greater learning slopes compared to vehicle-treated animals (p = 0.01) (Fig. 5B), indicating improved learning ability in CN-105 mice after ICH.

### Neuroinflammation was reduced in CN-105 treated mice

**Figure 6:**
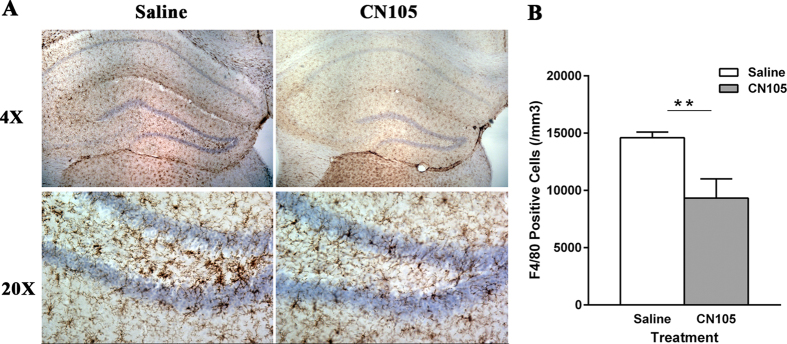
CN-105 reduces brain microglial activation after ICH. (**A**) Representative images of F4/80-labelled microglia in ipsilateral hippocampi at 5 days after ICH in mice treated with vehicle or CN-105. (**B**) Stereological analysis showed that CN-105 treatment significantly decreased the number of F4/80-positive cells in the ipsilateral hippocampi. ** *p* < 0.01, compared to vehical group, n = 3–4.

Neuroinflammation was measured by immunohistochemistry with an anti-mouse F4/80 antibody, a marker of macrophage/microglia. Since most part of striatum with collagenase was damaged by blood, the adjacent hippocampal area was chosen for stereological analysis. At 5 days after ICH, the number of F4/80-positive cells in the ipsilateral hippocampi was significantly decreased in CN-105 treated mice compared to vehicle-treated animals (9327 ± 838 vs 14593 ± 116 cells/mm^3^, p = 0.003) (Fig. 6B). Further, morphological changes of microglia were observed with smaller sized cell body and thinner processes in CN-105 mice compared to vehicle group (Fig. 6A), indicating CN-105 reduces microglial activation.

### Neuronal survival was improved in CN-105 mice

**Figure 7:**
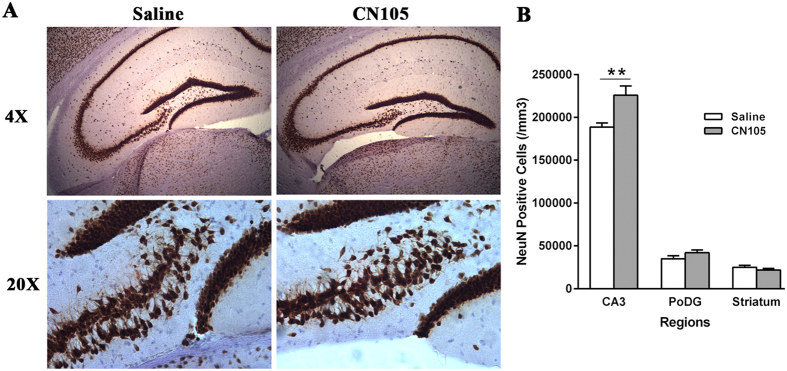
Hippocampal CA3 neuronal survival increased in CN-105 mice, based on NeuN staining and stereology at 33 days post ICH. NeuN-positive cells were counted in ipsilateral CA3 and dentate gyrus polymorphic region of dorsal hippocampus and striatum (**A**). Ipsilateral NeuN-positive cell numbers were significantly increased in the CA3 region of the dorsal hippocampus after injury, compared to vehicle-treated counterparts (**B**). (**p < 0.01; n = 7/group).

Immunohistochemistry and unbiased stereology showed significantly increased NeuN-positive cell numbers in the CA3 region (225773 ± 10841 vs 188719 ± 4780 cells/mm^3^, p = 0.009), but not in the dentate gyrus polymorphic region (42109 ± 8439 vs 34919 ± 9378 cells/mm^3^, p = 0.16) or striatum (21816 ± 5073 vs 25142 ± 5777 cells/mm^3^, p = 0.27) in the CN-105 vs the vehicle group (Fig. 7).

## Discussion

CN-105, a pentapeptide derived from the polar binding face of the apoE receptor binding region, easily cross BBB, reduces gliosis and cerebral edema, and improves functional outcomes and neuronal survival in a preclinical model of murine ICH.

Treatment of ICH remains a compelling area of unmet medical need, as no effective therapeutic strategies are available. Following initial injury, brain inflammatory responses mediate the development of cerebral edema, intracranial hypertension, and secondary neuronal injury. Currently, medical management is directed toward reducing hemorrhage volume via intensive blood pressure control, correcting pre-existing coagulopathy, optimizing cerebral perfusion, and treating intracranial hypertension caused by the hematoma and surrounding edema.

Although outcomes after ICH are generally poor, there is a wide degree of variability that is likely mediated by genetic influences. For example, apoE polymorphism appears to play a significant role in determining outcomes following ICH, as the apoE4 isoform is associated with increased cerebral edema and neurologic morbidity. This genetic association sparked research on the isoform-specific role of apoE in mediating outcome following ICH and other acute brain injuries.

Several earlier studies found that endogenous apoE may also exert direct neuroprotective effects by reducing NMDA-mediated excitotoxicity^21^,^22^,^23^. In addition, our lab and others have demonstrated that apoE modifies neuroinflammatory responses in an isoform-specific manner through its adaptive role in the downregulation of glial activation^12^,^24^,^25^,^26^. The mechanism of this action is driven by specific interaction between the receptor binding region of apoE (residues 130–149) and glial cell surface receptors, such as low-density lipoprotein receptor-related protein 1 (LRP1)^22^,^27^. LRP1 is a member of the scavenger class of receptors, which selectively binds oxidatively modified lipids. Since LRP1 would be activated in an inflammatory CNS environment where myelin and lipids are exposed to oxidative stress, it is plausible that it plays an adaptive role by downregulating secondary neuroinflammatory responses and mitigating secondary neuronal injury^28^. Recent data suggest that increased affinity of the apoE3 protein isoform for the LRP1 receptor, which is present on both glial and neuronal cells, may explain its isoform-specific effects^29^.

The intact apoE holoprotein is too large to cross the blood-brain barrier (BBB), and thus has limited therapeutic potential when administered peripherally. To address this limitation, smaller apoE peptides ranging from 12–20 amino acids were created. These were derived from the receptor-binding region of apoE, and share the immunomodulatory properties of the intact holoprotein and downregulate brain inflammatory responses *in vitro* and *in vivo*. Further, apoE peptides are small enough to cross the BBB, are well tolerated, and improve outcomes in a variety of preclinical models of acute brain injury. There is strong data suggesting that endogenous apoE, and apoE mimetic peptides reduce neuroinflammatory responses and reduce secondary tissue injury. In cell culture models, the effect of apoE on reducing neuroinflammatory responses was first described in 1997^24^, and the demonstration that apoE mimetic petides retained this function in murine and human cell line was described in 2001^26^. The effect of endogenous apoE in suppressing neuroinflamatory responses in our murine model of TBI^30^,^31^,^32^, and the effect of apoE mimetic peptides in reducing CNS inflammation and ameliorating tissue injury *in vivo* was subsequently described^12^,^15^. Indeed, functional and histological outcomes are improved when these peptides are administered after ICH^8^,^9^,^11^, traumatic brain injury^13^,^15^,^32^,^33^, subarachnoid hemorrhage^10^,^15^, focal stroke^14^,^34^, and experimental allergic encephalomyelitis^35^,^36^. This demonstrates then, that the reduction in neuroinflammation and excitotoxicity affected by apoE peptides is associated with sustained functional improvements in these preclinical acute brain injury models. These experiments also demonstrate the therapeutic potential of small peptides in acute brain injury and neurological disorders characterized by glial activation and neuroinflammatory responses.

More recently, we created small 5-amino acid peptides that mimic the polar face of the apoE helical domain involved in receptor interactions. In our initial non-GLP PK studies the pentapeptide CN-105 demonstrated approximately 5 fold higher than prior apoE based mimetic peptides^15^ The present study tested the therapeutic value of pentapeptide CN-105 in a murine model of collagenase-induced ICH, and found that it was neuroprotective and improved both short-term and long-term functional outcomes after ICH. Importantly, pentapeptide CN-105 is 10 times more potent than prior apoE-based peptides, eg, COG 1410, in similar preclinical models^9^,^11^.

These findings are noteworthy, but some study limitations must be recognized. Collagenase-induced ICH model was used because it replicates deep basal ganglia hemorrhage with hemorrhage expansion that is seen clinically. However, introducing this bacterial collagenase may result in a greater inflammatory response than might otherwise be present^37^. Thus, in future work, these findings should be replicated in other validated models of ICH, such as autologous blood injection. The therapeutic effect of CN-105 need to be investigated in other brain injury models including traumatic brain injury (TBI), subarachnoid hemorrhage (SAH) and ischemia. Of note, the receptor binding region of apoE does not contain the amino acid substitutions at positions 112 and 158 that define the different protein isoforms and modify binding to the LDL receptor family by allosteric effects, and thus are not an ideal model for studying isoform-specific effects of apoE. Also, peptide therapeutics often have a much shorter half-life than more traditional small-molecule therapies. However, although CN-105 was administered acutely, the pharmacodyanamic effects were durable and sustained over the 33-day testing period.

In conclusion we found that the small 5-amino acid base apoE-mimetic peptide CN-105 improved neurobehavioral outcomes, decreased edema, and increased neuronal survival over a 33-day testing period after preclinical ICH, but did not reduce hematoma volume or expansion. Therefore, CN-105 has significant potential for clinical translation as an acute ICH therapeutic, although future studies must attempt to replicate these findings in other ICH models and higher order animals.

## Methods

### Animals

All procedures were reviewed and approved by the Duke University Institutional Animal Care and Use Committee in keeping with established guidelines. Twelve- to 14-week-old male C57BL/6J mice (Jackson Laboratory, Bar Harbor, ME) were housed on a 12-hour light/dark cycle in standard acrylic cages with ad libitum access to food and water. In each experiment, mice were randomized to treatment or vehicle groups before injury. Animals were treated with blinded concealment. All procedures and assessments were performed in blinded fashion.

### Experimental cohorts

Experiment 1. CN-105 pharmacokinetics in Brain and Plasma. The concentration of CN-105 was determined at 1, 3, 10, 15, 30 and 60 minutes (n = 5/time point) post injection using LC/MS.

Experiment 2. Hematoma volume. Hemorrhage volume was assessed by magnetic resonance imaging (MRI) at 2 hours post ICH (before treatment) and 24 hours post ICH. 3 saline- and 3 CN-105-treated animals were perfused at 24 hours post ICH, and brain hematoxylin and eosin (H&E) staining was performed. CN-105 or saline was given by tail vein injection at 2, 6, and 24 hours post-ICH.

Experiment 3. Brain water content. Brain water content was assessed at 24 hours post ICH in 8 saline- and 8 CN-105-treated animals. CN-105 or saline was given by tail vein injection at 2, 6, and 24 hours post ICH. Two animals randomized to CN-105 died at 4 and 6 hours post-ICH, and were excluded from further analyses.

Experiment 4. Neurobehavioral assessment. Animals were randomized to vehicle (n = 14) or CN-105 (n = 14) groups, and evaluated for neurobehavioral function (neuroscore), vestibulomotor function (rotarod), and spatial learning (MWM). Mice received tail vein injections of vehicle or CN105 at 4, 8, and 24 hours post ICH.

Experiment 5. Microglial staining. A separate cohort of animals was randomized to vehicle (n = 4) or CN-105 (n = 4) groups and received tail vein injections at 4, 8, and 24 hours post ICH. Immunohistochemical microglial staining was performed and gliosis was quantified by stereology. One mice in vehicle group died within 1 day after injury and was excluded.

Experiment 6. Neuronal survival. The hippocampus has been associated with spatial learning and memory. To determine whether histological outcome correlates with functional outcomes, neuronal density was quantified in the hippocampus and striatum of the mice (n = 7/group) in experiment 4 by stereology at 33 days post-ICH following MWM testing.

### ICH model

Intrastriatal collagenase injection was used to induce ICH in mice, as previously described^38^. Briefly, the trachea was intubated after anesthesia induction with 4.6% isoflurane, and the lungs were mechanically ventilated with 1.5% isoflurane in a mixture of 30%/70% O2/N2. Rectal temperature was maintained at 37 °C ± 0.2 °C by circulating warm water in an underbody waterbed. The animal’s head was secured in a stereotactic frame, and a midline scalp incision was made. After exposing the skull, a burr hole was created 2.2 mm left lateral to bregma, and a 0.5 μL syringe needle (Hamilton, Reno, NV, USA) was advanced to a depth of 3 mm from cortex. Type IV-S Clostridial collagenase (Sigma, St. Louis, MO, USA) was injected over 2 minutes (0.075 U in 0.4 μL normal saline). After closing the incision, animals were allowed to recover spontaneous ventilation, and were then extubated and given free access to food and water.

### Peptide synthesis and administration

CN-105 (Ac-VSRRR-amide) was synthesized by Polypeptide Inc. (San Diego, CA) to a purity of >99%, and was dissolved in normal saline. Using a murine model of traumatic brain injury, 0.05 mg/kg CN-105 was previously found to be the lowest dose that improved functional performance after injury (data not shown). Here, therefore, 100 μL 0.05 mg/kg CN-105 in saline, or vehicle, was administered via intravenous tail vein injection 3 times at pre-specified timepoints after ICH. Animals were gently restrained by a rodent restrainer (Harvard Apparatus, Holliston, MA) during each administration.

### CN105 Pharmacokinetics

A protocol was developed in collaboration with the Duke University Core Proteomics and Metabolomics Shared Resource using LC/MS on a high-resolution accurate-mass instrument. Recovery was optimized via the addition of urea to the homogenization buffer (suggesting tight binding to protein target in the intracellular matrix), and cleaned up using Oasis HLB extraction. After extraction, samples were dried down in Speed Vac and reconstituted in 25 uL of 1% ACN/0.1% TFA/0.02% HFBA and analyzed using a nanoAcquity LC (Waters) coupled to a Synapt G2 Q-ToF (Waters). Quantitative data was extracted as selected ion chromatograms for both the native ((M + 2H)^2+^at m/z 357.7 and heavy-labeled form CN-105 at m/z 362.7). Quantitation was accomplished by using a ratio of the peak area of the analyte and internal standard which was fit to a 7-point calibration curve (plasma R^2^ = 0.997 and brain R^2^ = 0.999).

### Magnetic resonance imaging

MR images were collected using a Bruker 7.0T MRI scanner (Bruker Biospin, Billerica, MA, USA) operating with Paravision 5.1. Animals were anesthetized with 1.5% isoflurane in room air. Core body temperature was maintained at 37 °C ± 0.5 °C by a circulating water bath, and respiratory rates were maintained at 50–70 respirations per second. Susceptibility Weighted Images (SWI) were collected using FLASH sequence with the following parameters: TE/TR = 9/700 ms, Flip Angle(FA) = 40, FOV = 3 cm x 3 cm, Matrix size = 256 × 256, thickness = 0.5 mm, slices = 32; Total scan time = 27 mins. SWI images were reconstructed using Paravison5.1 (Bruker Biospin Billerica MA USA). The DICOM images were post-processed in the off-line using OSIRIX imaging (www.osirixviewer.com) software to measure ICH volumes.

### Histological hemorrhage volume measurement

Histological hematoma volume was measured at 24 hours after injury to coincide with maximum hematoma expansion. After anesthesia, mice were euthanized by decapitation, and brains were immediately harvested and flash-frozen at −20 °C. Coronal sections (20 μm) were taken at 400-μm intervals over the rostral-caudal extent of the lesion. Sections were stained with hematoxylin and eosin. Lesion volume was measured by digitally sampling stained sections with an image analyzer (M2 Turnkey System, Imaging Research, Inc., St. Catharines, Ontario, Canada). Lesion volumes (mm^3^) were computed as running sums of lesion area multiplied by the known interval between sections (400 μm) over the extent of the lesion, and expressed as an orthogonal projection.

### Brain water content

Mice were anesthetized and euthanized at 24 hours after ICH to coincide with the point of maximum inflammatory changes. Brains were dissected and sectioned mid-sagittally, and cerebellum and brainstem were removed. Each hemisphere was then weighed immediately (‘wet’ weight), and re-weighed after dehydrating for 24 hours at 105 °C (‘dry’ weight). Cerebral edema was expressed as water content, calculated as a percentage of wet weight ([wet weight - dry weight]/[wet weight] x 100).

### Neurological score

An observational rating system^8^ was used to evaluate neurobehavioral function before ICH (baseline) and on Days 1–5 after ICH to assess early changes. This rating system uses a 4-point scale (0–3) with a maximum total of 21 points to evaluate basic neurobehavioral status in 7 functional domains: spontaneous activity, motor function, climbing, balance and coordination, body proprioception, vibrissae response, and tactile response. Lower scores denote more severe injury.

### Rotarod testing

An automated rotarod (Ugo Basile, Comerio, Italy) was used to assess the effects of therapeutic intervention on vestibulomotor function^19^. On the day before injury, mice underwent 2 consecutive conditioning trials at a set rotational speed of 16 revolutions/minute for 60 seconds, followed by 3 additional trials at accelerating rotational speeds. The average time lapse to fall from the rotating cylinder in the second set of trials was recorded as baseline latency. To assess early motor outcome, mice underwent rotarod testing on Days 1–5 after injury. On each day, mice underwent 3 trials with an inter-trial interval of 15 minutes. Average latency to fall from the rod was recorded.

### Morris water maze testing

The MWM^20^ was used to assess the effects of therapeutic intervention on spatial learning and memory. It was conducted in a black aluminum pool (105 cm across, 60 cm deep) filled with 22 °C water opacified with powdered milk. A platform 7.5 cm in diameter was submerged 1 cm below the water surface, and 4 different visual signs were spaced equidistant on the wall of the pool at the same height above the water surface. The maze was kept in a room dedicated to behavioral testing, with light and sound unchanged throughout training and testing. Mice were tested on Days 29–32 after ICH to assess long-term memory. In a conditioning trial, each mouse was released into the pool for 90 seconds, guided to the hidden platform, and allowed to remain there for 15 seconds. On each of the 4 consecutive testing days, 4 trials were conducted with an inter-trial interval of 20–30 minutes. For each trial, mice were placed into the pool facing the perimeter, and were allowed to search for the platform for a maximum of 90 seconds. Mice that were not able to locate the platform within the allotted time were guided to it and allowed to remain there for 15 seconds, and were then returned to their heated home cage. Mice were started in one of 4 different quadrants for each trial, with starting quadrants randomly assigned each day. Latency to find the platform was recorded by a computerized video tracking system (KeilSoft LLC, Chapel Hill, NC).

### Immunohistochemistry and stereological analysis

After anesthetic induction, mice underwent transcardial perfusion with 30 mL normal saline. Brains were rapidly removed, immersion-fixed in 4% formaldehyde for 24 hours, and then transferred to 30% sucrose/1x PBS and stored at 4 °C for 48 hours. Frozen coronal sections (40 μm) were collected on a freezing sliding microtome. Floating brain sections were incubated in 1% hydrogen peroxide, permeabilized by 0.1% Saponin, and blocked with 10% goat serum. Sections were incubated overnight with an anti-mouse F4/80 antibody (a microphage/microglial marker, rat monoclonal, 1:10,000; Serotec, Raleigh, NC, USA), or a mouse monoclonal antibody, anti-neuronal-specific nuclear protein (NeuN, a neuronal marker, 1:90,000; Chemicon, Temecula, CA, USA). Biotinylated goat anti-rat or anti-mouse immunoglobulin G secondary antibody (1:3000; Vector Laboratories, Inc, Burlingame, CA) was then applied for 1 hour, followed by avidin-biotin-peroxidase complex treatment for 1 hour (ABC kit; Vector Laboratories, Inc.). Staining was visualized with diaminobenzidine (DAB kit; Vector Laboratories, Inc). After mounting onto slides, all sections were counterstained with hematoxylin (Fisher Scientific, Fair Lawn, NJ). Cells were counted using a Nikon 218912 light microscope interfaced with the StereoInvestigator software package (MicroBrightField, Williston, VT, USA). The number of stained cells per volume of the hippocampus (F4/80), or CA3 and dentate gyrus polymorphic regions of the hippocampus (NeuN), and the striatum (NeuN) was estimated by using an optical fractionator method, as previously described^18^,^39^.

### Statistical analyses

Repeated measures analysis of variance (RM-ANOVA) was used to compare neuroseverity scores, rotarod latencies, and MWM latencies, with time as the repeated variable. Bonferroni correction was used for the repeated measures technique in ANOVA. Student’s *t*-test was used to compare hematoma volume, brain water content, immunohistochemistry, and learning slopes in MWM. A *p* value < 0.05 was considered statistically significant. All values are expressed as means ± standard error. Statistical analysis was performed using StatView (SAS Institute Inc, Cary, NC, USA).

## Additional Information

**How to cite this article**: Lei, B. *et al*. Neuroprotective pentapeptide CN-105 improves functional and histological outcomes in a murine model of intracerebral hemorrhage. *Sci. Rep*. **6**, 34834; doi: 10.1038/srep34834 (2016).
