# Erratum: Neuroprotective pentapeptide CN-105 improves functional and histological outcomes in a murine model of intracerebral hemorrhage

**DOI:** 10.1038/srep39580

**Published:** 2017-01-05

**Authors:** Beilei Lei, Michael L. James, Ji Liu, Guanen Zhou, Talaignair N. Venkatraman, Christopher D. Lascola, Shawn K. Acheson, Laura G. Dubois, Daniel T. Laskowitz, Haichen Wang

Scientific Reports 6: Article number: 34834; 10.1038/srep34834 published online: 10 07 2016; updated: 01 05 2017.

The Competing Financial Interests statement in this Article should read:

“D.T.L. is an officer of Aegis CN, LLC, which supplied the CN-105”.

