# Author Correction: Neuroprotective pentapeptide CN-105 improves functional and histological outcomes in a murine model of intracerebral hemorrhage

**DOI:** 10.1038/s41598-020-63178-2

**Published:** 2020-04-20

**Authors:** Beilei Lei, Michael L. James, Ji Liu, Guanen Zhou, Talaignair N. Venkatraman, Christopher D. Lascola, Shawn K. Acheson, Laura G. Dubois, Daniel T. Laskowitz, Haichen Wang

**Affiliations:** 1 Department of Anesthesiology, Duke University Medical Center, Durham, NC 27710 USA; 2 Department of Neurology, Duke University Medical Center, Durham, NC 27710 USA; 3 Department of Neurology, Huanhu Hospital, Tianjin, 300060 China; 4 Department of Radiology, Duke University Medical Center, Durham, NC 27710 USA; 5 Department of Psychiatry, Duke University Medical Center, Durham, NC 27710 USA; 6 Neurobiology Research Lab, Durham VA Medical Center, Durham, NC 27705 USA; 7 Proteomics and Metabolomics Shared Resource, Duke University, Durham, NC 27710 USA

Correction to: *Scientific Reports* 10.1038/srep34834, published online 07 October 2016

This Article contains errors in Figure 2A. The 24 hr SWI image for Saline is a duplication of the 2 hr SWI image for Saline, the correct 24 h SWI image for Saline is incorrectly displayed as the 2 hr SWI image for CN105, the correct 2 hr SWI image for CN105 is incorrectly displayed as the 24 hr SWI image for CN105, and the correct 24 hr SWI image for CN105 is missing. The errors in Figure 2A do not affect the quantification result shown in Figure 2B. The correct Figure 2 appears below as Fig. 1.

**Figure 1:**
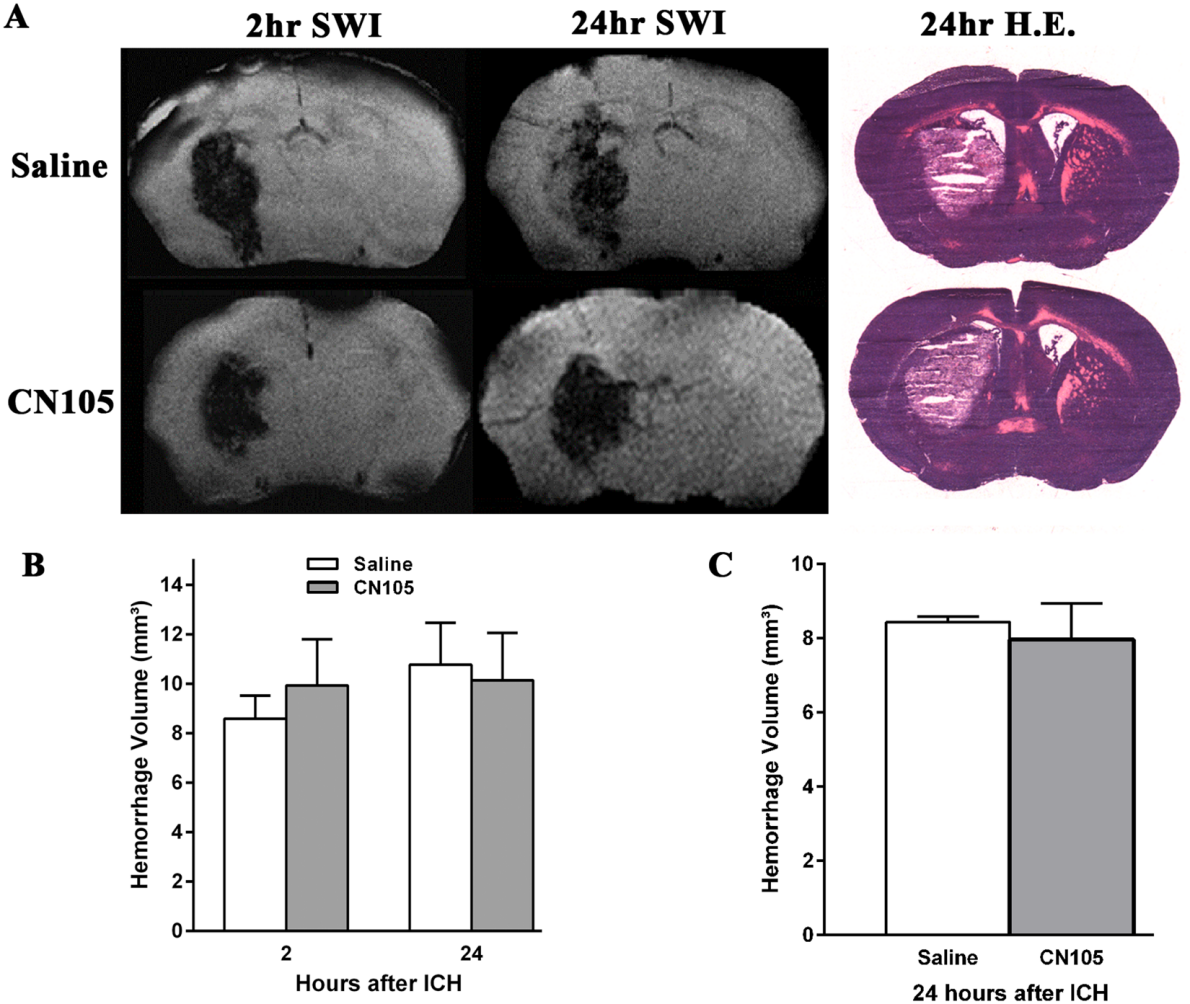
.

